# Xuebijing injection protects sepsis induced myocardial injury by mediating TLR4/NF-κB/IKKα and JAK2/STAT3 signaling pathways

**DOI:** 10.18632/aging.204990

**Published:** 2023-08-30

**Authors:** Xiang-Fei Kang, Xiao-Li Lu, Cheng-Fei Bi, Xiao-Dong Hu, Ying Li, Jin-Kui Li, Li-Shan Yang, Jia Liu, Lei Ma, Jun-Fei Zhang

**Affiliations:** 1 Department of Emergency Medical, General Hospital of Ningxia Medical University, Yinchuan 750000, Ningxia, China; 2 Medical Experimental Center, General Hospital of Ningxia Medical University, Yinchuan 750000, Ningxia, China; 3 School of Clinical Medicine, Ningxia Medical University, Yinchuan 750000, Ningxia, China; 4 Laboratory Animal Centre, Ningxia Medical University, Yinchuan 750000, Ningxia, China

**Keywords:** sepsis induced myocardial injury, Xuebijing, apoptosis, inflammation, JAK2/STAT3

## Abstract

Objective: Compelling evidence has demonstrated that Xuebijing (XBJ) exerted protective effects against SIMI. The aims of this study were to investigate whether TLR4/IKKα-mediated NF-κB and JAK2/STAT3 pathways were involved in XBJ's cardio-protection during sepsis and the mechanisms.

Methods: In this study, rats were randomly assigned to three groups: Sham group; CLP group; XBJ group. Rats were treated with XBJ or sanitary saline after CLP. Echocardiography, myocardial enzymes and HE were used to detect cardiac function. IL-1β, IL-6 and TNF-α in serum were measured using ELISA kits. Cardiomyocyte apoptosis were tested by TUNEL staining. The protein levels of Bax, Bcl-2, Bcl-xl, Cleaved-Caspase 3, Cleaved-Caspase 9, Cleaved-PARP, TLR4, p-NF-κB, p-IKKα, p-JAK2 and p-STAT3 in the myocardium were assayed by western blotting. And finally, immunofluorescence was used to assess the level of p-JAK2 and p-STAT3 in heart tissue.

Results: The results of echocardiography, myocardial enzyme and HE test showed that XBJ could significantly improve SIMI. The IL-1β, IL-6 and TNF-α levels in the serum were markedly lower in the XBJ group than in the CLP group (*p*<0.05). TUNEL staining's results showed that XBJ ameliorated CLP-induced cardiomyocyte apoptosis. Meanwhile, XBJ downregulated the protein levels of Bax, Cleaved-Caspase 3, Cleaved-Caspase 9, Cleaved-PARP, TLR4, p-NF-κB, p-IKKα, p-JAK2 and p-STAT3, as well as upregulated the protein levels of Bcl-2, Bcl-xl (*p* <0.05).

Conclusions: In here, we observed that XBJ's cardioprotective advantages may be attributable to its ability to suppress inflammation and apoptosis via inhibiting the TLR4/ IKKα-mediated NF-κB and JAK2/STAT3 pathways during sepsis.

## INTRODUCTION

Sepsis is a heterogeneous disease with life-threatening organ dysfunction and a high fatality rate that is brought on by a dysregulated host response to infection [1]. Around 20% of deaths worldwide yearly are attributed to sepsis [2], which continues to be the leading cause of death globally. Despite accumulated tough attempts involving rapid control of infection, hemodynamic stabilization and organ support to treat septic patients, clinical trials of intervention therapies have failed to yield promising results [3–5]. Sepsis induced myocardial injury (SIMI) is a common and severe complication of the multi-organ dysfunction followed by sepsis, although it is rarely the first to manifest. In the intensive care unit (ICU), septic cardiomyopathy continues to be a challenging obstacle to surmount; patients typically present with ventricular dilatation, decreased ventricular contractility, and right and left ventricular dysfunction [6, 7]. Of note, SIMI is the leading cause of death in ICU. A meta-analysis of clinical cases revealed that SIMI was significantly correlated with higher 1-month mortality in sepsis patients and increased in-hospital mortality in patients with hospital stays longer than 10 days [8]. Currently, a significant problem in the clinical management of sepsis is that there is no viable preventative and therapy method for SIMI. In order to find appropriate clinical treatment options, we need to better comprehend the pathophysiological mechanisms relating to the occurrence and management of SIMI.

Apoptosis is thought to play an important role in sepsis-induced organ failure and immune dysregulation [9, 10]. Previous researches on SIMI have confirmed the cardio-protection effects of anti-apoptosis in sepsis, too [11, 12]. As a result, it is imperative that cardiomyocyte apoptosis is one element of the promising therapeutic strategies for patients with SIMI. It is shown that the activation of TLR4/NF-κB and JAK2/STAT3 pathways induced apoptosis and inflammation during sepsis [13]. Researches have also suggested that inhibiting the TLR4/NF-κB and JAK2/STAT3 pathways protects the heart in septic rat model [14, 15]. Therefore, resisting apoptosis by inhibiting the activation of TLR4/NF-κB and JAK2/STAT3 signaling pathways may have clinical benefits for patients who suffer from sepsis induced myocardial dysfunction.

**Figure 1:**
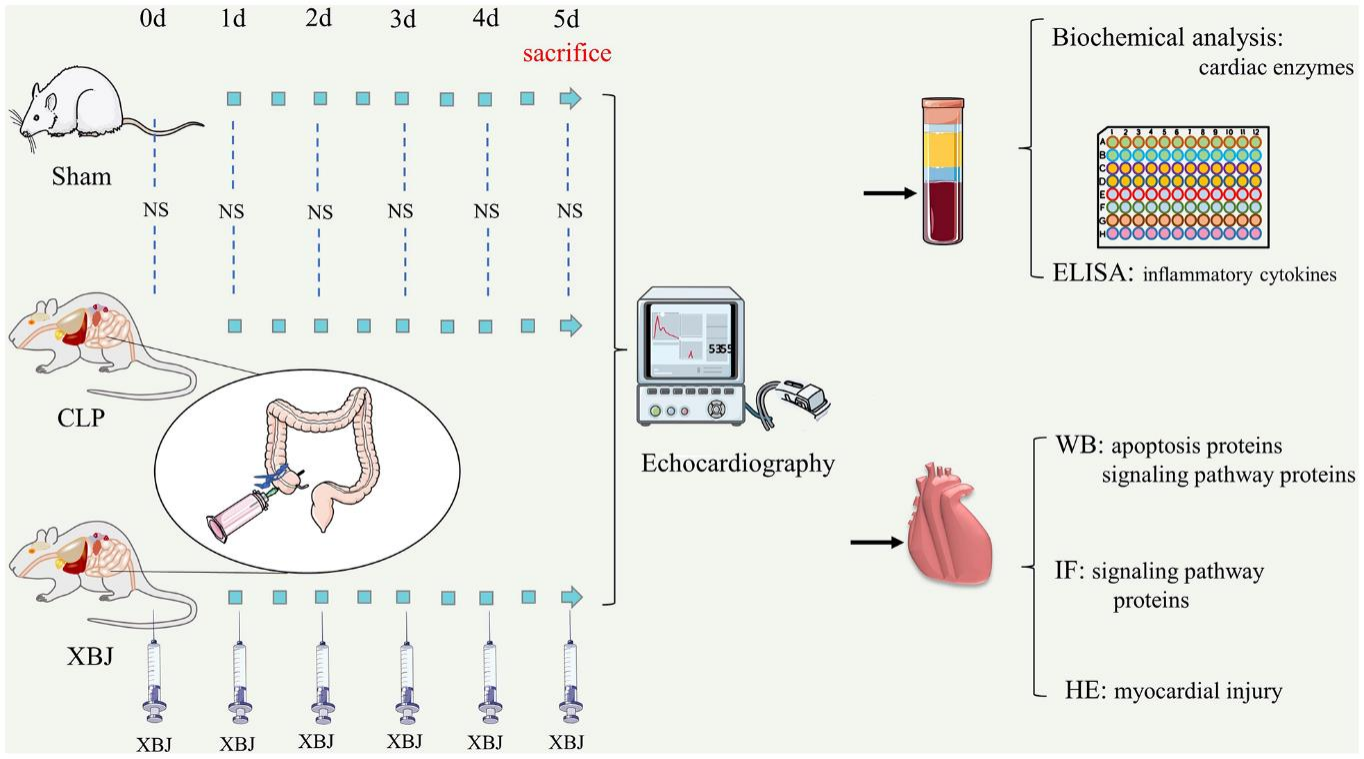
Workflow of the present study in a graphical manner.

The Chinese Food and Drug Administration (Beijing, China, Number Z20040033) approved XueBiJing (XBJ), a Chinese herbal remedy, in 2004 for the clinical treatment of sepsis [16]. The four primary biological functions of immunity, apoptosis, inflammation, and coagulation have been found in XBJ, a drug used to treat sepsis [17, 18]. In clinical settings, XBJ has been shown to improve pneumonia severity index and severe community-acquired pneumonia [19], as well as complications in patients with sepsis [16, 20]. Furthermore, XBJ was found to ameliorate the organ dysfunction caused by sepsis in the septic rat model [21, 22]. Currently, it can be established that XBJ has a potentially protective effect on SIMI [23]. However, it is unknown whether XBJ can prevent SIMI by blocking the activation of the TLR4/NF-B and JAK2/STAT3 pathways, which provoke apoptosis. Despite the fact that finding a cure for SIMI is a nearly insurmountable job, we have been striving towards one since the disease was discovered. In this study, we sought to explore whether XBJ influences apoptosis in septic rat exposed to CLP through the TLR4/IKKα-mediated NF-κB and JAK2/STAT3 pathways, which may be considered as a prospective molecular target for prevention and therapy of SIMI. The study workflow is illustrated in Figure 1.

## RESULTS

### XBJ alleviated the myocardial damage in CLP-induced sepsis rat model

**Figure 2:**
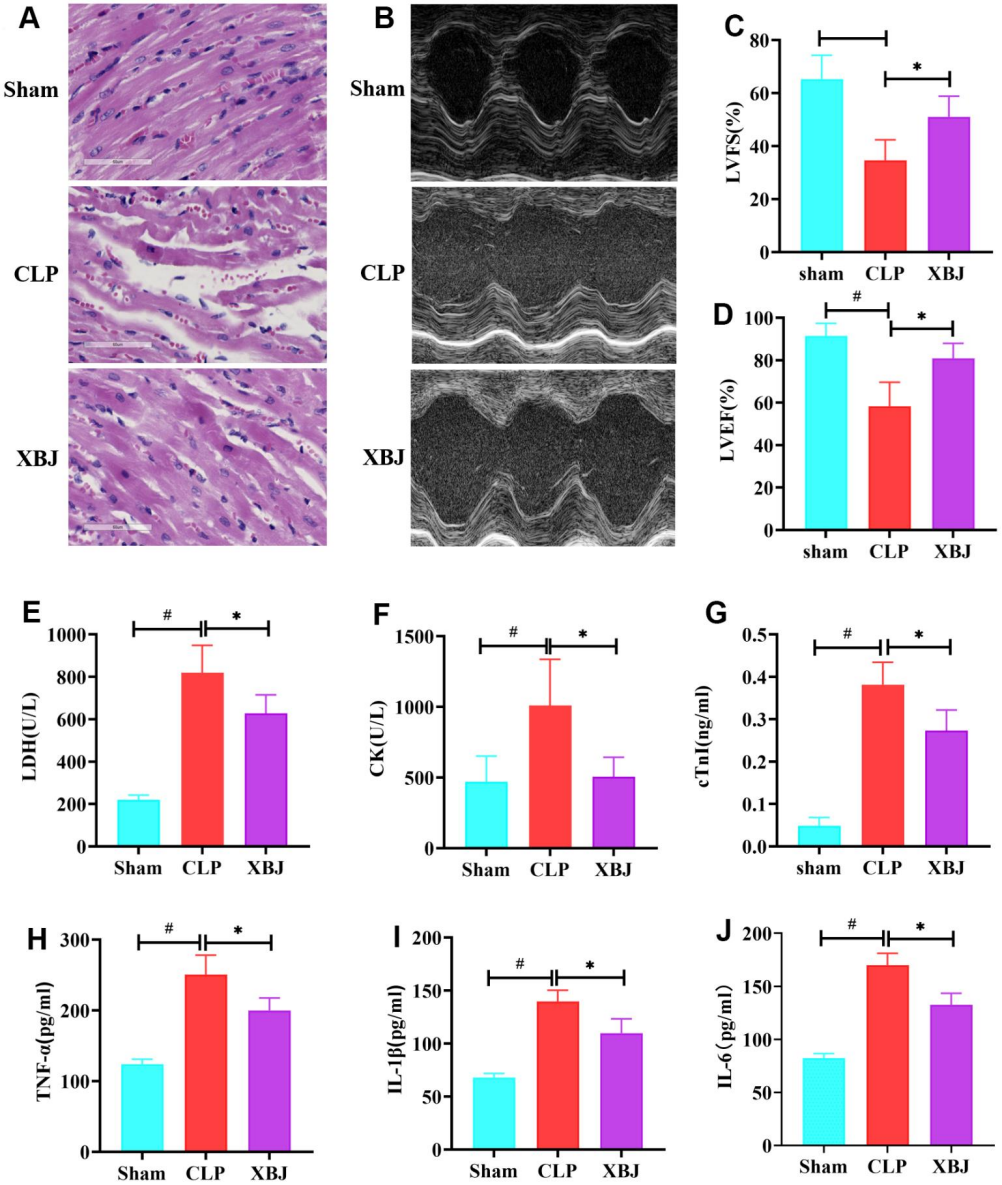
**Effects of XBJ on myocardial damage and inflammation in rat after CLP.** (**A**) Pathological changes of myocardial tissues under HE staining (200×). (**B**) Representative echocardiographic images. (**C**, **D**) Quantification of LVFS and LVEF via echocardiography. (**E**–**G**) XBJ improved myocardial dysfunction in rat after CLP: Serum levels of LDH (**E**), CK (**F**) and cTnI (**G**) detected by automated biochemical analyzer. (**H**–**J**) XBJ alleviated the myocardial inflammation in rat after CLP: Serum levels of TNF-α (**H**), IL-1β (**I**) and IL-6 (**J**) detected by ELISA. Data are expressed as mean ± SD (n = 6/group), # indicates *p*<0.05 (vs. Sham group), * indicates *p*<0.05 (vs. CLP group).

First, the cardiac functional state of CLP-induced sepsis rat model was evaluated by echocardiography (Figure 2B), and the results of the analysis were used to derive the two cardiac function indexes, LVFS (Figure 2C) and LVEF (Figure 2D), respectively. When compared to the Sham group, the CLP group's ejection fraction and shortening fraction were both significantly lower (*p*<0.05). While XBJ markedly enhanced cardiac function in the CLP-treated rat, as evidenced by increased LVFS and LVEF (*p*<0.05). In addition, HE staining of heart tissues was performed for exploring the role of XBJ in preventing the CLP-mediated myocardial injury. The CLP group had loose and light staining (interstitial edema) in the myocardium of the CLP-treated rat as opposed to the normal morphology of myocardial cells in the Sham group, and XBJ alleviated these pathological abnormalities in the myocardium of the CLP-treated rat (Figure 2A). To measure the concentrations of LDH (Figure 2E), CK (Figure 2F), and cTnI (Figure 2G) in rat serum, biochemical analysis was carried out. These three myocardial injury markers changed in a way that was consistent with the findings of the HE staining. As shown in our results, the LDH, CK, and cTnI levels in the serum were significantly higher in the CLP group than in the Sham group (*p*<0.05), whereas they were markedly lower in the XBJ group than in the CLP group (*p*<0.05). These results suggested that XBJ developed a protective role on myocardial damage in CLP-treated rat.

### XBJ reduced the inflammation in CLP-induced sepsis rat model

There are a lot of pro-inflammatory cytokines in the blood circulation when the body is subjected to sepsis. To assess the effect of XBJ on inflammatory responses in CLP-treated rat, the levels of TNF-α (Figure 2H), IL-1β (Figure 2I) and IL-6 (Figure 2J) in the peripheral blood were detected by ELISA. Relative to Sham group, CLP group had remarkable increased TNF-α, IL-1β and IL-6 serum levels, with the differences being of statistical significance (*p*<0.05). Furthermore, the serum levels of TNF-α, IL-1β, and IL-6 in the CLP-treated rat were remarkably decreased under the therapy of XBJ (*p*<0.05). These results revealed that XBJ reduced the excessive inflammatory response in CLP-treated rat.

### XBJ interacted with JAK2/STAT3 signaling pathway directly

**Figure 3:**
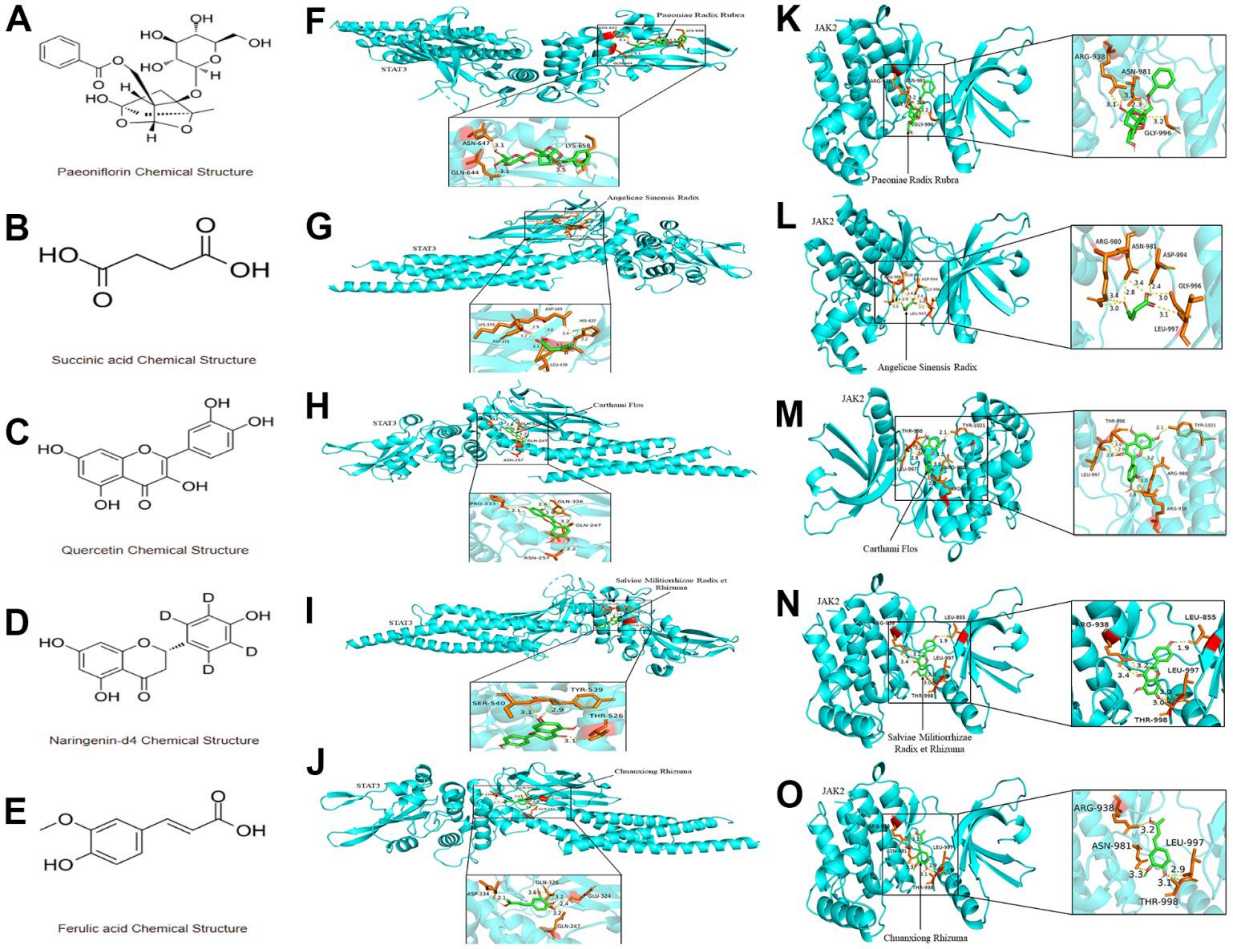
**The interaction between the five monomer components of XBJ and JAK2/STAT3 signaling pathway.** (**A**–**E**) The molecular structure of the monomers corresponding to the five components of XBJ: paeoniflorin (**A**), Succinic acid (**B**), Quercetin (**C**), Naringenin (**D**) and Ferulic acid (**E**). (**F**–**O**) 3D crystal structure for paeoniflorin-JAK2 complex (**F**), Succinic acid-JAK2 complex (**G**), Quercetin-JAK2 complex (**H**), Naringenin-JAK2 complex (**I**), Ferulic acid-JAK2 complex (**J**), paeoniflorin-STAT3 complex (**K**), Succinic acid-STAT3 complex (**L**), Quercetin-STAT3 complex (**M**), Naringenin-STAT3 complex (**N**), and Ferulic acid-STAT3 complex (**O**).

XBJ injection is known to have five key pharmacological components: *Paeoniae Radix Rubra, Angelicae Sinensis Radix, Carthami Flos, Salviae Militiorrhizae Radix et Rhizuma and Chuanxiong Rhizuma*. We selected the monomers corresponding to these five pharmacological components for molecular docking with JAK2/STAT3 signaling pathway through the Autodock Vina docking procedure. As our docking results suggested, the binding energies of complexes for paeoniflorin-JAK2, Succinic acid-JAK2, Quercetin-JAK2, Naringenin-JAK2, Ferulic acid-JAK2, paeoniflorin-STAT3, Succinic acid-STAT3, Quercetin-STAT3, Naringenin-STAT3, and Ferulic acid-STAT3 were -7.9 kcal/mol, -4.2 kcal/mol, -7.4 kcal/mol, -8.3 kcal/mol, -6.3 kcal/mol, -6.9 kcal/mol, -4.2 kcal/mol, -7.3 kcal/mol, -7.7 kcal/mol and -5.9 kcal/mol, respectively. We also presented the molecular structure of the monomers corresponding to the five components of XBJ in Figure 3A–3E. Furthermore, the 3D binding conformational structure for the paeoniflorin-JAK2 complex showed that a total of 3 hydrogen bonds were formed of paeoniflorin with ARG-938, ASN-981 and GLY-996 of JAK2 (Figure 3F). There are 5 hydrogen bonds that were formed of Succinic acid with ARG-980, ASN-981, ASP-994, GLY-996 and LEU-997 of JAK2 in the 3D crystal structure for the Succinic acid-JAK2 complex (Figure 3G). There are 5 hydrogen bonds that were formed of Quercetin with THR-998, TYR-1021, ARG-980, ARG-938 and LEU-997 of JAK2 in the 3D crystal structure for the Quercetin-JAK2 complex (Figure 3H). There are 4 hydrogen bonds that were formed of Naringenin with ARG-938, LEU-855, LEU-997 and THR-998 of JAK2 in the 3D crystal structure for the Naringenin-JAK2 complex (Figure 3I). There are 4 hydrogen bonds that were formed of Ferulic acid with ARG-938, LEU-997, THR-998 and ASN-981 of JAK2 in the 3D crystal structure for the Ferulic acid-JAK2 complex (Figure 3J). There are 3 hydrogen bonds that were formed of paeoniflorin with ASN-647, LYS-658 and GLN-644 of STAT3 in the 3D crystal structure for the paeoniflorin-STAT3 complex (Figure 3K). There are 5 hydrogen bonds that were formed of Succinic acid with LYS-370, ASP-369, HIS-437, LEU-438 and ASP-371 of STAT3 in the 3D crystal structure for the Succinic acid-STAT3 complex (Figure 3L). There are 4 hydrogen bonds that were formed of Quercetin with PRO-333, GLN-326, GLN-247 and ASN-257 of STAT3 in the 3D crystal structure for the Quercetin- STAT3 complex (Figure 3M). There are 3 hydrogen bonds that were formed of Naringenin with SER-540, TYR-539 and THR-526 of STAT3 in the 3D crystal structure for the Naringenin-STAT3 complex (Figure 3N). There are 4 hydrogen bonds that were formed of Ferulic acid with ASP-334, GLN-326, GLU-324 and GLN-247 of JAK2 in the 3D crystal structure for the Ferulic acid-JAK2 complex (Figure 3O). In this study, JAK2 and STAT3's protein-ligand binding sites were found in the inhibitor-binding domain. These results supported the interaction between XBJ and JAK2/STAT3 signaling pathway.

### XBJ inhibited the expression of phosphorylated JAK2 and STAT3 proteins in CLP-induced sepsis rat model

**Figure 4:**
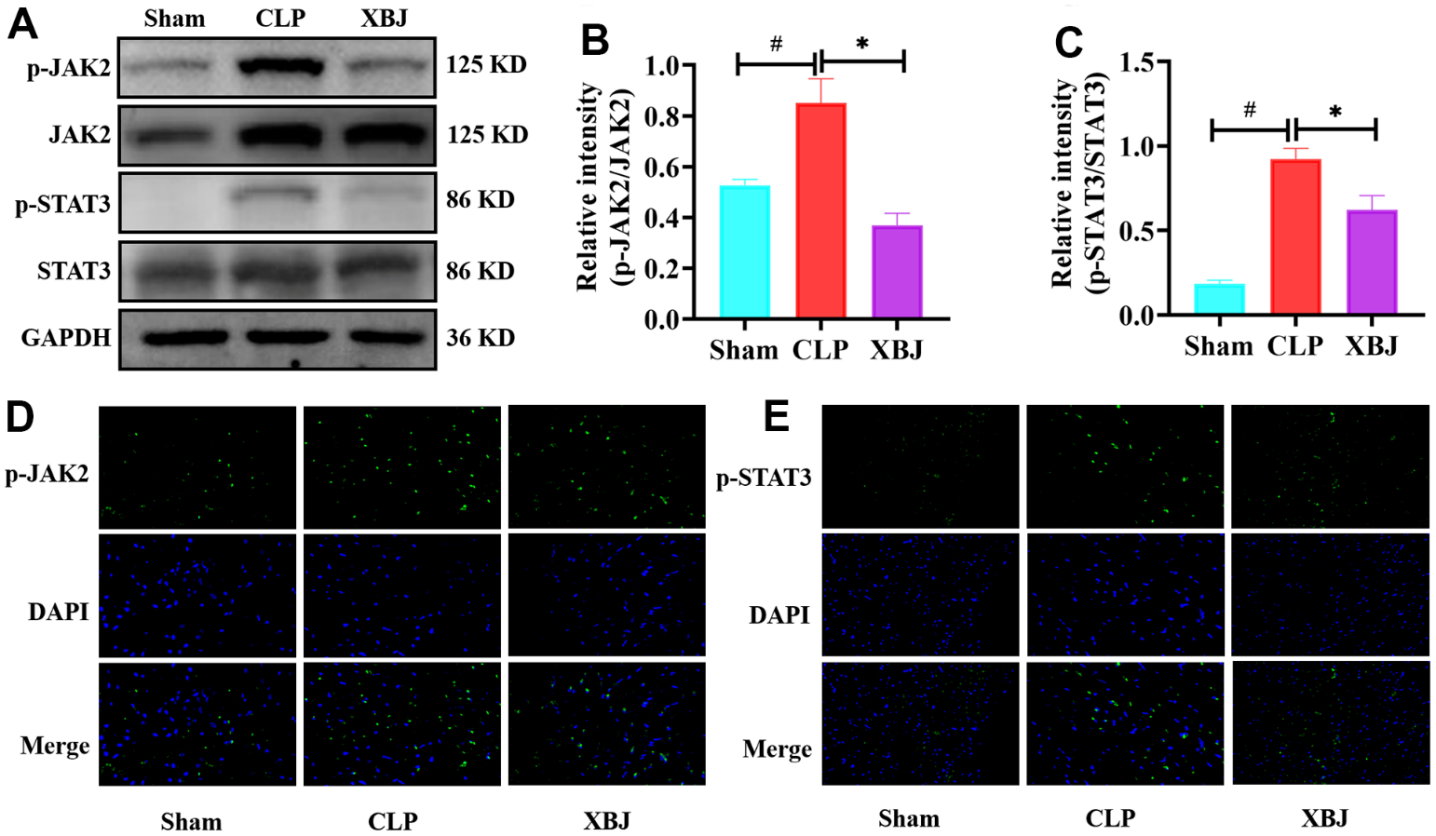
**Effects of XBJ on JAK2/STAT3 signaling pathway.** (**A**) Representative images of western blotting for p-JAK2, JAK2, p-STAT3 and STAT3 *in vivo* (GAPDH was used as the internal reference protein). (**B**, **C**) Relative intensity of p-JAK2/JAK2 (**B**) and p-STAT3/STAT3 (**C**) was analyzed by western blotting. (**D**, **E**) Representative images of the protein expressions of p-JAK2 and p-STAT3 in myocardial tissue were detected by immunofluorescence localization. Positive p-JAK2 (**D**) and p-STAT3 (**E**) cells were stained green, with the sections counterstained with DAPI to visualize nuclei (blue). Data are expressed as mean ± SD (n=3/group), # indicates *p*<0.05 (vs. Sham group), * indicates *p*<0.05 (vs. CLP group).

After verifying that XBJ protects against cardiac injury, and excessive inflammation in rat after CLP condition, we looked into how XBJ influences the activation of JAK2/STAT3 pathways. On the one hand, western blotting was performed to detect phosphorylation levels of proteins in the JAK2/STAT3 pathways. p-JAK2 (Figure 4A, 4B), and p-STAT3 (Figure 4A, 4C) expression levels in the CLP group were obviously higher than those in the Sham group, however the application of XBJ blocked the high expression (*p*<0.05). On the other hand, immunofluorescence was subjected to pinpoint where the JAK2/STAT3 pathway was phosphorylated in the heart tissue. p-JAK2 (Figure 4D) and p-STAT3 (Figure 4E) mainly accumulated on cardiomyocyte in the CLP group, whereas the pretreatment with XBJ lessened their levels (*p*<0.05). These results demonstrated that XBJ repressed the phosphorylation levels of JAK2/STAT3 pathways in CLP-treated rat.

### XBJ inhibited the expression of phosphorylated TLR4, NF-κB and IKKα proteins and suppressed the cardiomyocyte apoptosis in CLP-induced sepsis rat model

**Figure 5:**
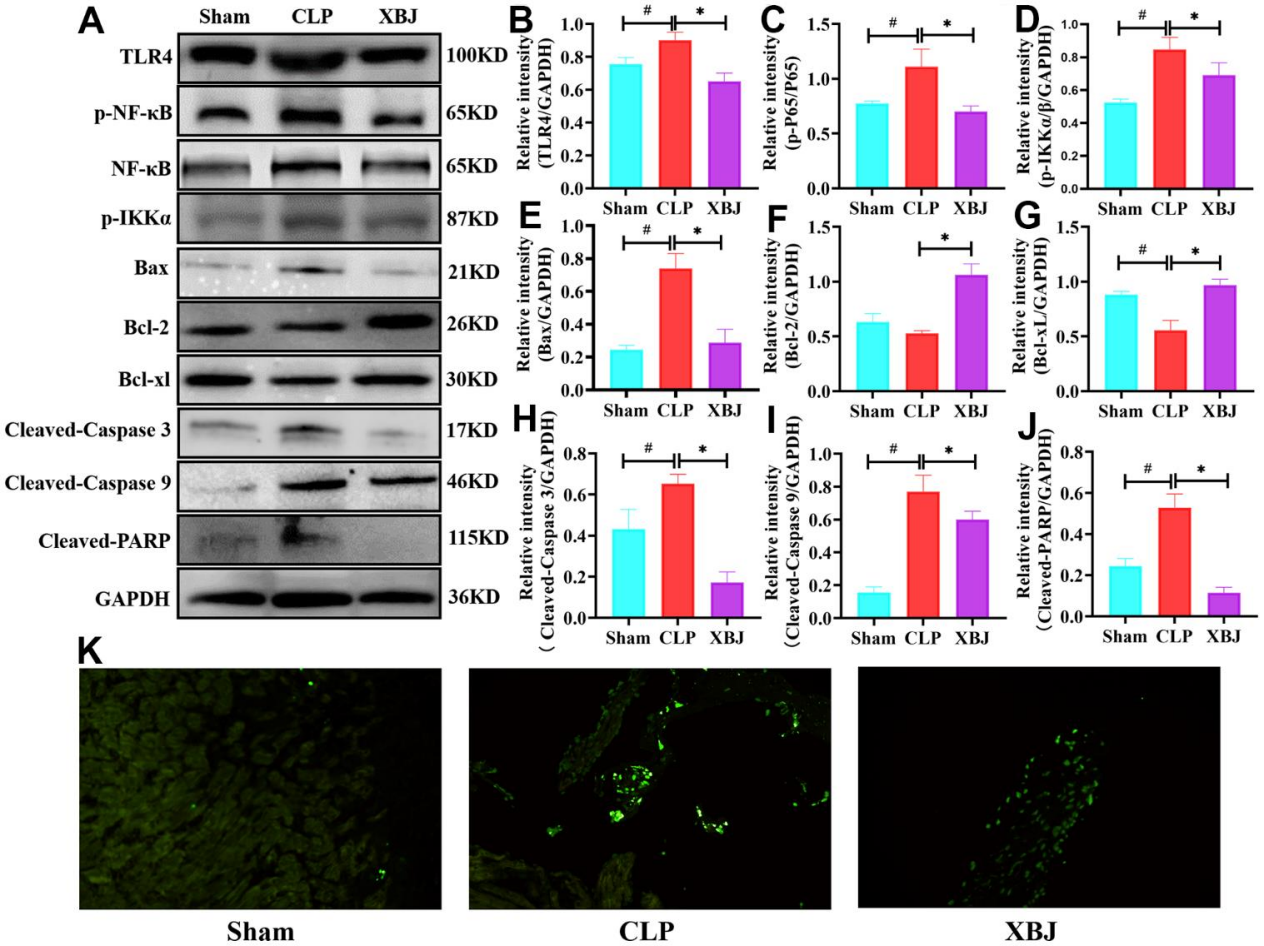
**Effects of XBJ on apoptosis-related proteins and TLR4/NF-κB/IKKα signaling pathway.** (**A**) Representative images of western blotting for TLR4, p-NF-κB, NF-κB, p-IKKα, Bax, Bcl-2, Bcl-xl, Cleaved-Caspase 3, Cleaved-Caspase 9 and Cleaved-PARP *in vivo* (GAPDH was used as the internal reference protein). (**B**–**J**) Relative intensity of TLR4 (**B**), p-NF-κB/NF-κB (**C**), p-IKKα (**D**), Bax (**E**), Bcl-2 (**F**), Bcl-xl (**G**), Cleaved-Caspase 3 (**H**), Cleaved-Caspase 9 (**I**) and Cleaved-PARP (**J**) was analyzed by western blotting. Data are expressed as mean ± SD (n=3/group), # indicates *p*<0.05 (vs. Sham group), * indicates *p*<0.05 (vs. CLP group). (**K**) Representative images show apoptosis of heart tissue was detected with TUNEL staining.

We looked further into how XBJ influences the activation of TLR4/NF-κB/IKKα pathways. Western blotting was performed to detect phosphorylation levels of proteins in the TLR4/NF-κB/IKKα pathways. TLR4 (Figure 5A, 5B), p-NF-κB (Figure 5A, 5C) and p- IKKα (Figure 5A, 5D) expression levels in the CLP group were obviously higher than those in the Sham group, however the application of XBJ blocked the high expression (*p*<0.05). Activation of apoptosis plays an important role in myocardial injury in sepsis. The expression of proapoptotic proteins (Bax, Cleaved-Caspase 3, Cleaved-Caspase 9, and Cleaved-PARP) and antiapoptotic proteins (Bcl-2 and Bcl-xl) was tested using western blotting to evaluate the impact of XBJ on CLP-mediated cardiomyocyte apoptosis. In the CLP group, apoptosis-related proteins Bax (Figure 5A, 5E), Cleaved-Caspase 3 (Figure 5A, 5H), Cleaved-Caspase 9 (Figure 5A, 5I), and Cleaved-PARP (Figure 5A, 5J) had dramatically higher expression levels, but Bcl-2 (Figure 5A, 5F) and Bcl-xl (Figure 5A, 5G) had sharply lower expression levels (*p*<0.05). However, injection of XBJ reversed changes of these apoptosis-related proteins in the myocardium of CLP-treated rat (*p*<0.05). Additionally, we used TUNEL staining (Figure 5K) to observe that the CLP group's cardiomyocyte apoptosis was clearly apparent when compared to the myocardial structure of the Sham group. However, treatment with XBJ significantly improved CLP-induced cardiomyocyte apoptosis. These results indicated that XBJ suppressed the cardiomyocyte apoptosis brought on by CLP in rat.

## DISCUSSION

**Figure 6:**
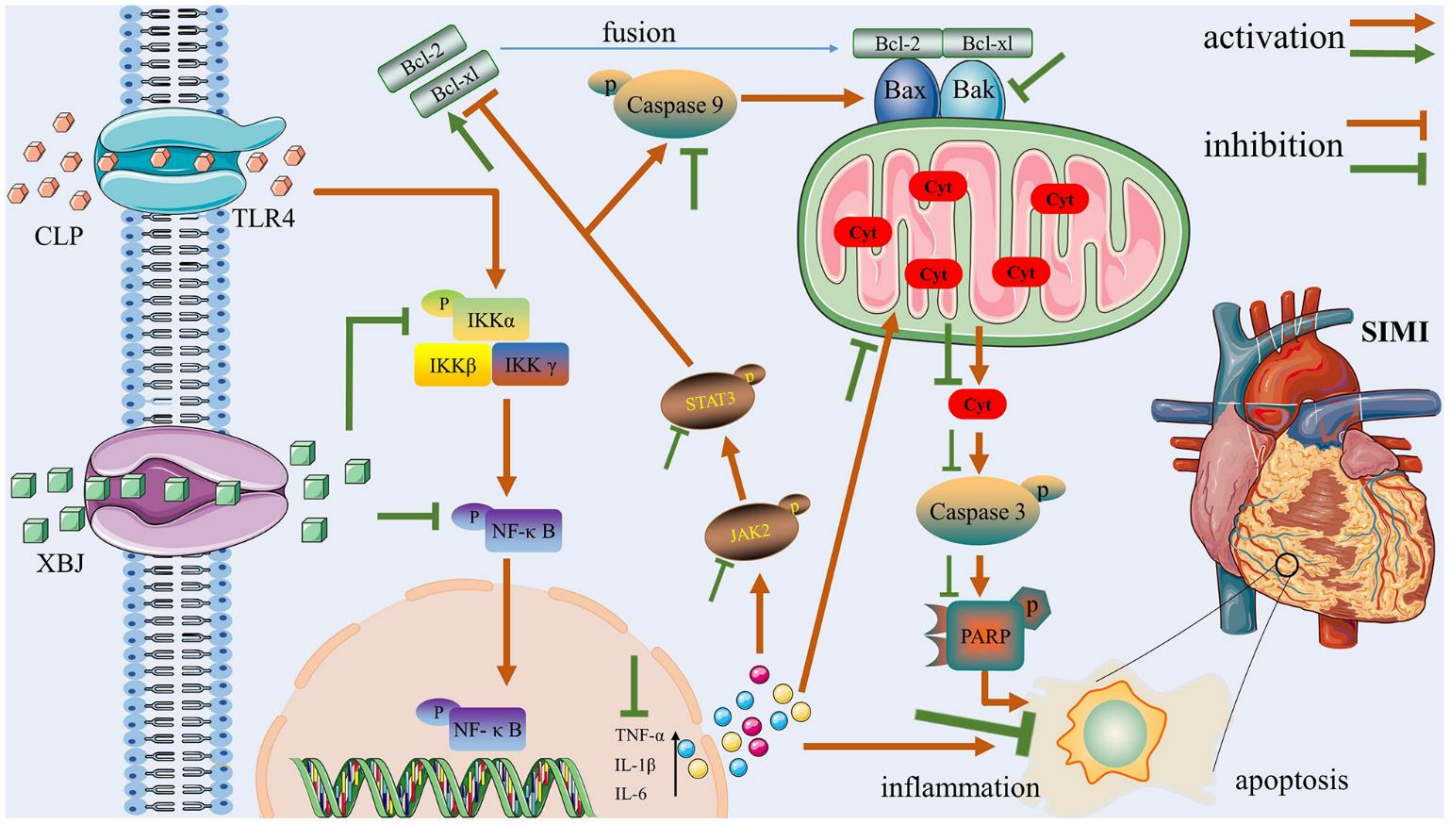
The potential protective mechanisms of XBJ in SIMI caused by CLP.

One of the main illnesses that represent a grave hazard to people's health all around the world is sepsis, a potentially lethal host reaction to infection that results in organ failure. Myocardial injury is one of the most common side effects and a key factor of fatalities in septic patients. It was reported that apoptosis and inflammatory storms have a role in the pathophysiological mechanisms of SIMI [24]. Compelling evidence from clinical or basic research has demonstrated that XBJ exerted protective effects against sepsis. So, we designed this experiment to explore the underlying mechanisms of XBJ protects against SIMI. CLP is known to be the gold standard for simulating sepsis models in *in vivo* experiments [25]. In our study, we induced sepsis in rats with CLP to mimic the clinical context of patients with sepsis. There is one highlight: The protective effect of XBJ on SIMI may play an anti-apoptotic and anti-inflammatory role by regulating TLR4/IKKα-mediated NF-κB and JAK2/STAT3 signaling pathways. Figure 6 illustrates these three components, which will serve as the central concepts of this article.

Sepsis is an inflammatory condition that associates with the initialization of exogenous pathogen-associated molecular patterns (PAMPs) and endogenous damage-associated molecular patterns (DAMPs) [26]. Similarly, Potential candidates responsible for septic cardiomyopathy include PAMPs and DAMPs [27]. Furthermore, DAMPs may contribute to myocardial cell and tissue injury during sepsis [28]. The action of inflammatory factors (such as TNF-α, IL-1β, IL-6, etc.), mitochondrial dysfunction, reactive oxygen species mediated oxidative stress and cardiomyocyte apoptosis are some of the plausible explanations in the progression of SIMI [29–32]. The pathogenesis of SIMI is extremely intricate. By reviewing previous studies, Wang, Jie et al. proposed to classify cardiac dysfunction in sepsis patients based on hemodynamic alterations: left ventricular systolic insufficiency, left ventricular diastolic insufficiency, right ventricular systolic insufficiency, right ventricular diastolic insufficiency, or mixed cardiac alterations [33]. In this way, it seems like SIMI is characterized by a compromised left ventricular systolic function. The advantage of echocardiography is that it is relatively simple to assess cardiac function with noninvasive techniques [34]. So, to assess the status of rats' cardiac function, we choose to use LVEF in combination with LVFS. Consistent with previous study [35], our findings suggested that CLP-induced septic rats had significantly impaired left ventricular systolic function as evidenced by decreased LVEF and LVFS. Additionally, the cut-off threshold for aberrant LVEF has been established by numerous research to range between 40% and 50% [34]. In our study, the decrease of LVEF level in the CLP group was statistically significant when compared to the Sham group even though it was not below the lower limit of normal (50%). One theory to support this result is that, due to drastically diminished afterload during sepsis, when septic shock develops, LVEF can remain to be normal despite severely compromised intrinsic left ventricular contractility [36, 37]. Two XBJ components, paeoniflorin and hydroxy saffron yellow A, have reportedly been shown to lessen sepsis-caused cardiac dysfunction in previous research [38], which is in line with the results of the present study. Our data revealed that LVEF and LVFS dramatically increased after treatment with XBJ as compared to the CLP group, indicating that XBJ restored cardiac function in sepsis-affected rats. Taken together, XBJ was effective in preventing cardiac dysfunction brought on by sepsis.

Additionally, the pathophysiological processes of SIMI also involve injured cardiomyocytes and cardiomyocyte inflammatory storms. One of the gold standards for determining myocardial damage in clinical settings is the serological examination of cTn [39]. It has been proposed that cTn have the potential to assess cardiac function in patients with SIMI [40]. Serum cTnI is also a highly specific and sensitive biomarker of myocardial injury [41]. In addition to the feedback given by serum cTnI levels, the severity of myocardial injury is also reflected by the serum activity of cardiac enzymes. It is known that cardiac enzymes are mostly located in heart muscle cells. When heart muscle is injured, proteins from heart muscle cells termed cardiac enzymes, like those of AST, LDH, CK, and CK-MB, are released into the bloodstream [42]. Numerous studies have shown that increased LDH, CK, and cTnI concentrations may reflect not only ischemic heart disease but also myocardial injury in non-cardiogenic diseases such as sepsis [43–45]. Therefore, in this study, we detected the cardiac biomarkers LDH, CK, and cTnI in rat serum to determine sepsis-mediated myocardial damage. The activation of DAMPs in rats that indicates the structural damage to cardiac myocytes may be the reason why we discovered that serum levels of LDH, CK, and cTnI were significantly higher in rats after CLP; but when XBJ was administered to septic rats, the levels of LDH, CK, and cTnI in the serum greatly reduced, thereby lessening the myocardial injury. DAMPs perpetuate organ damage, the cytokine storm, and the inflammatory response in sepsis [46]. The potential of TNF-α, IL-1β, and IL-6 as biomarkers for patients with sepsis has been proposed, and the combined test of the three has an excellent predictive value in individuals with sepsis-induced cardiomyopathy [47, 48]. Several *in vivo* research have revealed that CLP is an intervention that induces the release of significant amounts of TNF-α, IL-1β, and IL-6 into the bloodstream [49, 50]. In our study, the same conclusion was drawn. These results suggest that sepsis-induced myocardial suppression may be closely related to the stimulation of inflammatory factors leading to cardiomyocyte inflammation. In addition, we discovered that XBJ treatment significantly decreased TNF-α, IL-1β, and IL-6 serum levels in septic rat when compared to the CLP group. Jiang et al. demonstrated that XBJ had an anti-inflammatory impact in sepsis [22], which accounts for the discrepancy in our data between the CLP and XBJ groups. Thus, we hypothesize that XBJ's suppression of myocardial cell inflammatory outbreak may be responsible for its improvement in SIMI.

Apoptosis is a major step of sepsis-induced multiple organ failure, which is involved in the development and progression of lethal sepsis [51]. One of the main features in the pathophysiological process of SIMI is the sepsis-induced cardiomyocyte apoptosis. According to existing reports, myocardial apoptosis has been observed in CLP-induced sepsis models with pathophysiological changes of cardiac dysfunction and myocardial injury [11, 35, 52]. Bax, a member of the Bcl-2 family proteins, is a pro-apoptotic protein. A relatively new hypothesis regarding apoptosis was put forth in a recent article: forcible interplay of Bax with DRP1 causes it to relocate to the mitochondria, accumulate in apoptotic foci, activate the permeability of the mitochondrial outer membrane, and undergo mitochondrial remodeling that results in apoptosis [53]. Furthermore, Cytochrome c is released from mitochondria when Bax is located there, which triggers the induction of apoptosis [54]. Bcl-2 and Bcl-xl, other members of the Bcl-2 family proteins, are anti-apoptotic proteins. When activated Bax and Bak's BH3 structural domains are bound by anti-apoptotic proteins, heterodimers are created that decrease the permeability of the mitochondrial membrane and prevent apoptosis [55]. The primary mediator of programmed cell death is caspases. Foremost, caspase-3 is a death protease that is regularly activated and catalyzes the precise cleavage of numerous essential cellular proteins [56]. Of note, Caspase-3 is activated and triggers apoptosis under the direct control of apoptotic stimulators or the indirect control of mitochondrial cytochrome c release [56]. As an upstream signal for Caspase-3, Caspase-9 is activated by stimulation of cytochrome C and apoptotic vesicles [57]. Interestingly, Caspase-9 is required to activate Bax, which induces apoptosis during infection [58]. The apoptotic process is similarly mediated by Caspase-3's cleavage of PARP [59]. *In vivo* studies have proven the cardioprotective role of anti-apoptosis during sepsis and highlighted the engagement of Bax, Bcl-2, Bcl-xl, Caspase 3, and Caspase 9 in sepsis-induced cardiomyocyte apoptosis [11, 60]. In rat cardiac tissue following CLP, we noticed high expression of Cleaved-Caspase 3, Cleaved-Caspase 9, and Cleaved-PARP and low expression of Bcl-2 and Bcl-xl. XBJ treatment, however, prevented this myocardial apoptosis. Therefore, XBJ's suppression of myocardial cell apoptosis also may be responsible for its improvement in SIMI. Although we discovered that sepsis-mediated cardiomyocyte damage may be mostly caused by inflammation and apoptosis in the present study, the regulatory mechanisms need to be further investigated.

Nuclear factor-kappa B (NF-κB)-regulated downstream genes have been found, such as cytokines, chemokines, and immune receptors that regulate apoptosis, immune cell differentiation, proliferation, antioxidant stress response, and signal transduction [61–63]. It is well discussed that the regulation of NF-κB pathway and how this pathway is involved in immune response and as the potential therapeutic target for inflammation-related disease and cancers [64]. In recent years, a growing number of studies have found that the TLR4/NF-B signaling pathway is involved in the pathogenic mechanisms of sepsis [65, 66]. NF-κB is known to exhibit pro-apoptotic effects in certain stimulated cell environments [67]. Inhibition of cardiomyocyte apoptosis mediated by NF-κB signaling pathway has a potential protective effect on sepsis induced cardiomyopathy [68]. Additionally, it has been suggested that the surge of inflammatory response during sepsis is dependent on the activation of NF-κB signaling pathway [50]. TLRs are expressed by cells of the innate immune system, monitor and recognize a variety of different disease-associated molecular patterns (PAMPs), and are the body's initial barrier against infectious diseases. TLR4 is a transmembrane protein that recognizes Gram-negative lipopolysaccharide (LPS). The LPS/TLR4 signaling pathway may induce massive inflammation and lead to sepsis [69]. The absence of TLR4, a classic upstream target of phosphorylated NF-κB signaling pathway, ameliorates sepsis-induced organ damage by downregulating activated inflammatory and apoptosis-associated proteins [70]. The TLR4-mediated NF-κB signaling pathway has been extensively reported to be involved in the pathophysiological mechanisms of SIMI [14, 71]. Phosphorylation of IKKα is critical for activation of the non-canonical NF-κB signaling pathway [64]. Meanwhile, phosphorylated IKKα signal has the potential to regulate the canonical NF-κB signaling pathway. In addition, the activation of TLR4-mediated NF-κB signaling pathway was suggested to be positively correlated to inflammation and apoptosis during sepsis induced organ dysfunction [13]. As a result, it is crucial to emphasize the potential molecular mechanisms of the TLR4/IKKα-mediated NF-κB signaling pathway that may be involved in the inflammatory response and apoptosis during the pathophysiology of sepsis. Finally, the efficacy and pharmacological mechanisms of XBJ for the treatment of sepsis have been demonstrated, with the anti-inflammatory effects of XBJ on sepsis through targeting the TLR4/NF-κB signaling pathway being particularly well elaborated [20]. And XBJ can protect cardiac function from CLP challenges [38]. Thus, the findings revealed that XBJ can mitigate SIMI by suppressing the TLR4/IKKα mediated NF-κB signaling pathway, which is in accordance with our data.

Janus kinase 2 (JAK2)/signal transducer and the activator of transcription 3 (STAT3) pathway are widely engaged in inflammation, immune regulation, cell differentiation, apoptosis and proliferation [72–74]. After acute or chronic systemic inflammation, the JAK2/STAT3 pathway is closely associated with regeneration of the heart, lung, liver, and kidney tissues in lipopolysaccharide (LPS)-induced mice [75]. In addition, the JAK2/STAT3 pathway plays a role in the onset and progression of sepsis [76]. JAK2, a member of the intracellular non-receptor tyrosine kinase family, transmits signals for cytokine production via the JAK2/STAT3 signaling pathway. The JAK2/STAT3 signaling pathway mediates IL-6 during sepsis and delivers it to particular effector cells, which are ultimately destroyed [77]. STAT3 is a transcriptional activator that responds to cytokines and growth factors and plays a key role in many cellular processes, such as cell growth and apoptosis [78]. Previous studies have shown that the activated JAK2/STAT3 signaling pathway can trigger the release of pro-inflammatory and pro-apoptotic proteins during sepsis [13, 74]. The inactivation of JAK2/STAT3 signaling pathway is a therapeutic target for sepsis through regulating inflammation [79]. In the cardiac tissue of septic rat exposed to CLP, the JAK2/STAT3 signaling pathway was frequently highly upregulated together with cardiomyocyte apoptosis and inflammation [15]. Our study demonstrates that JAK2/STAT3 signaling is essential for the development of SIMI, as evidenced by the high expression of p-JAK2 and p-STAT3 in CLP group. Also, as previously mentioned, XBJ can improve sepsis via reducing cardiomyocyte apoptosis and inflammation. Hence, it’s well established that XBJ can mitigate SIMI by blocking the activation of JAK2/STAT3 signaling pathway from this study.

The research presents a few constraints in addition to above results. Initially, we examined the efficacy of XBJ for SIMI at a single time point (at 5d after CLP); however, whether this is the most appropriate time span for therapy is still up for debate. Furthermore, as XBJ has a variety of pharmacological effects, it is yet unknown if there are any other mechanisms that could shield it from SIMI caused by CLP. Lastly, our experimental model mostly uses adult rats (6-8 weeks), whereas clinical patients with sepsis have a wide range of ages and are primarily elderly. As a result, our findings may only provide certain tips for the treatment of young clinical patients.

## CONCLUSIONS

In here, we enriched the potential molecular mechanisms of XBJ in SIMI. In our study, XBJ was observed to improve cardiac function and myocardial injury in rats suffering from CLP challenge, which was associated with inhibition of cardiomyocyte apoptosis and inflammation. Meanwhile, our findings elucidate the underlying mechanisms of XBJ on sepsis-induced cardiomyocyte apoptosis and inflammation based on NF-κB and JAK2/STAT3 pathways. Our experiment once again demonstrated the clinical value of XBJ in the application of SIMI from the molecular level. However, rat’s CLP-incurred sepsis model can’t completely simulate the clinical condition of patients. So, the positive treatment of XBJ for SIMI in our study needs further experimental investigations associated with clinical trials.

## MATERIALS AND METHODS

### Animals

All animal procedures followed the guidelines established by the Ningxia Medical University Animal Protection Committee and were approved by the General Hospital of Ningxia Medical University's Ethics Committee (Yinchuan, China). Adult (6 - 8 weeks) male Sprague Dawley (SD) rats (weight 220 ± 20 g) were purchased from and housed at the Ningxia Medical University laboratory animal center. Rats were raised in plastic cages with temperature and humidity-controlled room (22.8 ± 2.0° C and 50% ~ 60%, respectively) with a 12/12 hours light/dark cycle for 7 days to adjust to the environment. The rats were kept in groups (3 per cage), supplied tap water, and supplied a standard rat chow diet, unlimited. All experimental procedures in this study were in accordance with the National Institutes of Health's Guide for the Care and Use of Laboratory Animals.

### Molecular docking

The main monotone components of each drug in XBJ were selected for molecular docking. First, Succinic acid, Quercetin, Ferulic acid, paeoniflorin and Naringenin of the 2D structure were obtained from the PubChem database (https://pubchem.ncbi.nlm.nih.gov/), then the 2D structure import Chem3D software for 3D structure, and saved as mol2 format, and used Autodock Vina docking procedure (http://autodock.scripps.edu/) into PDBQT format. Then, high-resolution protein crystal structures of JAK2 and STAT3 were selected as the ligand, and its PDB format was downloaded from the RCSB PDB database (https://www.rcsb.org/), imported PYMOL software to remove water molecules and heteromolecules, then imported Autodock Vina software to add hydrogen atoms and saved as pdbqt format. Finally, with the drug structure as ligand and the protein structure as receptor, the docking box was formed by using Autodock Vina software and the results with the lowest docking energy were saved. PYMOL software was used to visualize the docking results with the lowest energy.

### Experimental protocols

Eighteen rats were randomly divided into 3 groups (n = 6/group): (1) Sham group; (2) CLP group; (3) XBJ group. Before the experiment, all rats were fasted for 12 hours. Subsequently, 40 mg/kg of 4% phenobarbital was given intraperitoneally to make the rats unconscious. The lower quadrant of the rats' abdomens was then shaved with an electric razor, cleaned with iodine volt, and placed on a special rat fixation plate (supine posture) before the experimental surgery towel was set out. No ligation or puncture of the intestine was performed on the rats in the Sham group; only a straightforward abdominal incision was made. For rats in the CLP and XBJ groups, a longitudinal skin incision was made along the abdominal white line with a scalpel, and a 3-4 cm incision was cut with surgical scissors to expose the abdominal cavity, and the cecum was separated using blunt dissecting forceps and removed, leaving the remaining small and large intestine in the peritoneal cavity. The cecum was ligated with 3-0 wire at 1/3 of the ileocecal flap, and a small amount of feces was extruded from it by puncturing the hole at the ligated end with an 18G needle to ensure patency. The cecum was then restored to its original position, and the peritoneum, rectus abdominis muscle and skin were closed layer by layer with 4-0 sutures. Postoperatively, all rats were placed on an electric blanket to maintain body temperature in the normal range.

### Drug treatment and sample collection

After surgery, all rats were kept alone and subcutaneously injected with lactated Ringer's solution (30 ml/kg) for fluid resuscitation. Imipenem/Cilastin (20 mg/kg s.c.) and flurbiprofen axetil injection (5 mg/kg i.v.) were used to alleviate postoperative pain. Rats in the XBJ group received XBJ (10 ml/kg s.c.) at 1 hour after CLP, whereas rats in the sham or CLP groups received the same amount of sterile saline treatment, with repeated dosing every 24 h for 5 days. The cardiac function was evaluated at 5 days after CLP. And then the serum and heart tissues were harvested and stored at -80° C for further investigation.

### Echocardiography

The cardiac function was evaluated at 5 days after CLP. The hair on the chest of the rats was removed using hair removal cream before they were put in the horizontal position after being anesthetized with 40 mg/kg of 4% phenobarbital. Echocardiographic images were recorded using the Ultra High Resolution Small Animal Ultrasound Imaging System (Vevo®2100 Imaging System, Visualsonics, Toronto, Canada) with a 15-MHz transducer. Parameters of cardiac function were measured on the 2-D mode in the parasternal long-axis view prior to M-mode imaging positioned perpendicular to the interventricular septum and posterior left ventricular wall. Heart rate was measured over 3 consecutive cycles. The left ventricular ejection fraction (LVEF) and left ventricular fractional shortening (LVFS) parameters were calculated by the software of Vevo770TM imaging system. An investigator who was not familiar with the experiment carried out all of the procedures.

### Biochemical detection for assessment of cardiac function

Heart punctures were used to swiftly collect blood samples. The blood samples were centrifuged at 3,000 rpm for 10 minutes at room temperature after 30 minutes of coagulation to extract the serum. Serum concentrations of lactate dehydrogenase (LDH), creatine kinase (CK) and cardiac troponin I (cTnI) were detected by an Automatic Biochemical Analyzer (Bio Majesty JCA-BM6010, JEOL Ltd., Japan).

### Enzyme-linked immunosorbent assay (ELISA) for inflammatory cytokine

Inflammatory cytokines, tumor necrosis factor-α (TNF-α), interleukin-1β (IL-1β) IL-6 and in serum were measured using ELISA kits, according to the manufacturer’s instructions (BioSwamp, Wuhan, China). The concentrations of the cytokines were quantified by referring to standard curves.

### Histopathological (HE) staining

At 5 days after CLP, myocardial tissues from each group were collected and fixed for 48 hours at room temperature with 4% paraformaldehyde. The fixed cardiac tissues were sectioned into 4 μm-thick cross-sections after being dehydrated, paraffin-embedded. 4 μm sections were stained using a HE Staining Kit (Biotopped, Beijing, China), according to the manufacturer’s instructions. Morphological changes in myocardial tissues were stained with HE and observed at 200× magnification under a light microscope (Leica, USA).

### Immunofluorescence assay

Immunofluorescence was used to assess the level of p-JAK2 (Ser473) and p-STAT3 (Tyr607) in heart tissue. Fixed heart tissues were removed with 0.5% Triton X-100 for 20 min. Tissues were blocked with 5% BSA blocking solution for 60 min at room temperature, following by washing with PBS. The tissues were then incubated with p-JAK2 (cat. AF3024, 1:300) and p-STAT3 (cat. AF3293, 1:300) overnight at 4° C and further stained with Goat anti-Rabbit lgG H&L (AlexaFluor®594) (cat. ZF-0516, 1:100) secondary antibody. Afterwards, heart tissue was stained with 4′, 6-diamidino-2-phenylindole (DAPI, C0060, Solarbio, China) and observed with a fluorescence microscope (MF43-N, Mshot, China) to obtain representative fluorescence images.

### TUNEL staining

Extensive DNA degradation is the signature of the late stage of apoptosis. Visualization of apoptotic cardiomyocytes was performed on left ventricular tissue cross sections (4 μm thick) using One-step TUNEL Apoptosis Detection Kit (Beyotime, Beijing, China) and according to the manufacturer’s procedure. TUNEL staining changes in myocardial tissues were observed with a fluorescence microscope (MF43-N, Mshot, China) to obtain representative fluorescence images.

### Western blotting

The heart tissues added to RIPA lysis buffer spiked with protease inhibitors and phosphorylated protease inhibitors (Servicebio, Wuhan, China) are crushed by adding magnetic beads in fully automatic sample freezer grinder (JXFSTPRP-CL, Shanghai Jing Xin, China). The protein concentration was measured using a BCA protein assay kit (Omni-Easy, Shanghai, China). Equal amounts of protein (5 μg/μl, 10 μl per lane) were separated by 7.5-12.5% SDS-PAGE and were transferred onto PVDF membranes using Bio-Rad western blotting analysis apparatus (CAVOY, Beijing, China). The membranes were then blocked with 5% skim milk powder at room temperature for 2 h and incubated at 4° C overnight with antibodies against TLR4 (cat. AF7071, 1:1000), phosphorylated (p)-NF-κB (cat. AF2006, 1:1000), NF-κB (cat. AF5006, 1:1000), p-IKKα (cat. AF3013, 1:1000), Bax (cat. AF0120, 1:2000), Bcl-2 (cat. AF6139, 1:2000), Bcl-xl (cat. AF6414, 1:1000), Cleaved-Caspase 3 (cat. AF7022, 1:1000), Cleaved-Caspase 9 (cat. AF5240, 1:1000), Cleaved-PARP (cat. AF7023, 1:1000), GAPDH (cat. T0004; 1:10,000), p-JAK2 (cat. AF3024, 1:1000), JAK2 (cat. AF6022, 1:1000), p-STAT3 (cat. AF3293, 1:1000) and STAT3 (cat. AF6294, 1:1000), followed by incubation at room temperature for 1h with goat anti-rabbit secondary antibodies (cat. S001; 1:10,000) or goat anti-mouse secondary antibodies (cat. AS014; 1:10,000; Abclonal). Protein bands were detected with an enhanced chemiluminescence kit (KeyGen BioTECH, Jiangshu, China) using capturing light sources with an ultrasensitive multifunction imager (Amersham lmager 680RGB) and were semi-quantified using ImageJ software (Rawak Software, Inc., Germany).

### Statistical analysis

All values described in the text and figures are presented as mean ± standard deviation (SD). The Kaplan-Meier method was applied to assess survival followed by the log rank test. One-way analysis of variance (ANOVA) test was used to compare among multiple groups, followed by Tukey’s test after a homogeneity test for variance and Tamhane T2’s test after a heterogeneity test for variance. SPSS 24.0 software was used to analyze the data. *p*<0.05 in two-tailed testing was considered statistically significant.

## References

[1] Singer M, Deutschman CS, Seymour CW, Shankar-Hari M, Annane D, Bauer M, Bellomo R, Bernard GR, Chiche JD, Coopersmith CM, Hotchkiss RS, Levy MM, Marshall JC, et al. The Third International Consensus Definitions for Sepsis and Septic Shock (Sepsis-3). JAMA. 2016; 315:801–10. 10.1001/jama.2016.0287 26903338 PMC4968574

[2] Vandewalle J, Libert C. Sepsis: a failing starvation response. Trends Endocrinol Metab. 2022; 33:292–304. 10.1016/j.tem.2022.01.006 35181202

[3] Zhang YY, Ning BT. Signaling pathways and intervention therapies in sepsis. Signal Transduct Target Ther. 2021; 6:407. 10.1038/s41392-021-00816-9 34824200 PMC8613465

[4] Lelubre C, Vincent JL. Mechanisms and treatment of organ failure in sepsis. Nat Rev Nephrol. 2018; 14:417–27. 10.1038/s41581-018-0005-7 29691495

[5] Karakike E, Scicluna BP, Roumpoutsou M, Mitrou I, Karampela N, Karageorgos A, Psaroulis K, Massa E, Pitsoulis A, Chaloulis P, Pappa E, Schrijver IT, Frantzeskaki F, et al. Effect of intravenous clarithromycin in patients with sepsis, respiratory and multiple organ dysfunction syndrome: a randomized clinical trial. Crit Care. 2022; 26:183. 10.1186/s13054-022-04055-4 35717241 PMC9206755

[6] Beesley SJ, Weber G, Sarge T, Nikravan S, Grissom CK, Lanspa MJ, Shahul S, Brown SM. Septic Cardiomyopathy. Crit Care Med. 2018; 46:625–34. 10.1097/CCM.0000000000002851 29227368

[7] Martin L, Derwall M, Al Zoubi S, Zechendorf E, Reuter DA, Thiemermann C, Schuerholz T. The Septic Heart: Current Understanding of Molecular Mechanisms and Clinical Implications. Chest. 2019; 155:427–37. 10.1016/j.chest.2018.08.1037 30171861

[8] Lin YM, Lee MC, Toh HS, Chang WT, Chen SY, Kuo FH, Tang HJ, Hua YM, Wei D, Melgarejo J, Zhang ZY, Liao CT. Association of sepsis-induced cardiomyopathy and mortality: a systematic review and meta-analysis. Ann Intensive Care. 2022; 12:112. 10.1186/s13613-022-01089-3 36513882 PMC9748009

[9] Ma Y, Liu J, Liu H, Han X, Sun L, Xu H. Podocyte protection by Angptl3 knockout via inhibiting ROS/GRP78 pathway in LPS-induced acute kidney injury. Int Immunopharmacol. 2022; 105:108549. 10.1016/j.intimp.2022.108549 35086056

[10] Zhao X, Zhang S, Shao H. Dexpanthenol attenuates inflammatory damage and apoptosis in kidney and liver tissues of septic mice. Bioengineered. 2022; 13:11625–35. 10.1080/21655979.2022.2070585 35510377 PMC9275904

[11] Li X, Luo J, Li Y, Jia L, Li Y, Ye S, Liu L, Yu Y, Lu Y, Luan Y. Macrophage-Derived Exosomes in TLR9-/- Mice Ameliorate Sepsis-Induced Mitochondrial Oxidative Stress and Apoptosis in Cardiomyocytes. Oxid Med Cell Longev. 2022; 2022:5719974. 10.1155/2022/5719974 36225174 PMC9550441

[12] Zhu X, Sun M, Guo H, Lu G, Gu J, Zhang L, Shi L, Gao J, Zhang D, Wang W, Liu J, Wang X. Verbascoside protects from LPS-induced septic cardiomyopathy via alleviating cardiac inflammation, oxidative stress and regulating mitochondrial dynamics. Ecotoxicol Environ Saf. 2022; 233:113327. 10.1016/j.ecoenv.2022.113327 35203005

[13] Zhou Y, Xu W, Zhu H. CXCL8_(3-72)_ K11R/G31P protects against sepsis-induced acute kidney injury via NF-κB and JAK2/STAT3 pathway. Biol Res. 2019; 52:29. 10.1186/s40659-019-0236-5 31084615 PMC6513525

[14] Xu X, Rui S, Chen C, Zhang G, Li Z, Wang J, Luo Y, Zhu H, Ma X. Protective effects of astragalus polysaccharide nanoparticles on septic cardiac dysfunction through inhibition of TLR4/NF-κB signaling pathway. Int J Biol Macromol. 2020; 153:977–85. 10.1016/j.ijbiomac.2019.10.227 31760017

[15] Zhen G, Liang W, Jia H, Zheng X. Melatonin relieves sepsis-induced myocardial injury via regulating JAK2/STAT3 signaling pathway. Minerva Med. 2022; 113:983–9. 10.23736/S0026-4806.20.06626-4 32683850

[16] Li C, Wang P, Zhang L, Li M, Lei X, Liu S, Feng Z, Yao Y, Chang B, Liu B, Shang H. Efficacy and safety of Xuebijing injection (a Chinese patent) for sepsis: A meta-analysis of randomized controlled trials. J Ethnopharmacol. 2018; 224:512–21. 10.1016/j.jep.2018.05.043 29860133

[17] Zhou W, Lai X, Wang X, Yao X, Wang W, Li S. Network pharmacology to explore the anti-inflammatory mechanism of Xuebijing in the treatment of sepsis. Phytomedicine. 2021; 85:153543. 10.1016/j.phymed.2021.153543 33799226

[18] Lv J, Guo X, Zhao H, Zhou G, An Y. Xuebijing Administration Alleviates Pulmonary Endothelial Inflammation and Coagulation Dysregulation in the Early Phase of Sepsis in Rats. J Clin Med. 2022; 11:6696. 10.3390/jcm11226696 36431172 PMC9694218

[19] Song Y, Yao C, Yao Y, Han H, Zhao X, Yu K, Liu L, Xu Y, Liu Z, Zhou Q, Wang Y, Ma Z, Zheng Y, et al. XueBiJing Injection Versus Placebo for Critically Ill Patients With Severe Community-Acquired Pneumonia: A Randomized Controlled Trial. Crit Care Med. 2019; 47:e735–43. 10.1097/CCM.0000000000003842 31162191 PMC6727951

[20] Li C, Wang P, Li M, Zheng R, Chen S, Liu S, Feng Z, Yao Y, Shang H. The current evidence for the treatment of sepsis with Xuebijing injection: Bioactive constituents, findings of clinical studies and potential mechanisms. J Ethnopharmacol. 2021; 265:113301. 10.1016/j.jep.2020.113301 32860891

[21] Liu J, Wang Z, Lin J, Li T, Guo X, Pang R, Dong L, Duan M. Xuebijing injection in septic rats mitigates kidney injury, reduces cortical microcirculatory disorders, and suppresses activation of local inflammation. J Ethnopharmacol. 2021; 276:114199. 10.1016/j.jep.2021.114199 33989736

[22] Jiang Y, Zou L, Liu S, Liu X, Chen F, Liu X, Zhu Y. GC/MS-based metabonomics approach reveals effects of Xuebijing injection in CLP induced septic rats. Biomed Pharmacother. 2019; 117:109163. 10.1016/j.biopha.2019.109163 31238257

[23] Zhang H, Wei L, Zhao G, Liu S, Zhang Z, Zhang J, Yang Y. Protective effect of Xuebijing injection on myocardial injury in patients with sepsis: a randomized clinical trial. J Tradit Chin Med. 2016; 36:706–10. 10.1016/s0254-6272(17)30003-1 29949330 PMC7147202

[24] Bi CF, Liu J, Yang LS, Zhang JF. Research Progress on the Mechanism of Sepsis Induced Myocardial Injury. J Inflamm Res. 2022; 15:4275–90. 10.2147/JIR.S374117 35923903 PMC9342248

[25] Bastarache JA, Matthay MA. Cecal ligation model of sepsis in mice: new insights. Crit Care Med. 2013; 41:356–7. 10.1097/CCM.0b013e318270e3ee 23269150 PMC3734801

[26] Moriyama K, Nishida O. Targeting Cytokines, Pathogen-Associated Molecular Patterns, and Damage-Associated Molecular Patterns in Sepsis via Blood Purification. Int J Mol Sci. 2021; 22:8882. 10.3390/ijms22168882 34445610 PMC8396222

[27] Kakihana Y, Ito T, Nakahara M, Yamaguchi K, Yasuda T. Sepsis-induced myocardial dysfunction: pathophysiology and management. J Intensive Care. 2016; 4:22. 10.1186/s40560-016-0148-1 27011791 PMC4804632

[28] Gotts JE, Matthay MA. Sepsis: pathophysiology and clinical management. BMJ. 2016; 353:i1585. 10.1136/bmj.i1585 27217054

[29] Lv X, Wang H. Pathophysiology of sepsis-induced myocardial dysfunction. Mil Med Res. 2016; 3:30. 10.1186/s40779-016-0099-9 27708836 PMC5037896

[30] Wang Y, Jasper H, Toan S, Muid D, Chang X, Zhou H. Mitophagy coordinates the mitochondrial unfolded protein response to attenuate inflammation-mediated myocardial injury. Redox Biol. 2021; 45:102049. 10.1016/j.redox.2021.102049 34174558 PMC8246635

[31] Merx MW, Weber C. Sepsis and the heart. Circulation. 2007; 116:793–802. 10.1161/CIRCULATIONAHA.106.678359 17698745

[32] Li N, Zhou H, Wu H, Wu Q, Duan M, Deng W, Tang Q. STING-IRF3 contributes to lipopolysaccharide-induced cardiac dysfunction, inflammation, apoptosis and pyroptosis by activating NLRP3. Redox Biol. 2019; 24:101215. 10.1016/j.redox.2019.101215 31121492 PMC6529775

[33] Wang J, Wang XT, Liu DW, Zhang HM, Su LX. Induction and deduction in sepsis-induced cardiomyopathy: five typical categories. Chin Med J (Engl). 2020; 133:2205–11. 10.1097/CM9.0000000000000929 32881720 PMC7508431

[34] Hollenberg SM, Singer M. Pathophysiology of sepsis-induced cardiomyopathy. Nat Rev Cardiol. 2021; 18:424–34. 10.1038/s41569-020-00492-2 33473203

[35] Han D, Li X, Li S, Su T, Fan L, Fan WS, Qiao HY, Chen JW, Fan MM, Li XJ, Wang YB, Ma S, Qiu Y, et al. Reduced silent information regulator 1 signaling exacerbates sepsis-induced myocardial injury and mitigates the protective effect of a liver X receptor agonist. Free Radic Biol Med. 2017; 113:291–303. 10.1016/j.freeradbiomed.2017.10.005 28993270

[36] Repessé X, Charron C, Vieillard-Baron A. Evaluation of left ventricular systolic function revisited in septic shock. Crit Care. 2013; 17:164. 10.1186/cc12755 23826739 PMC3706940

[37] Boissier F, Razazi K, Seemann A, Bedet A, Thille AW, de Prost N, Lim P, Brun-Buisson C, Mekontso Dessap A. Left ventricular systolic dysfunction during septic shock: the role of loading conditions. Intensive Care Med. 2017; 43:633–42. 10.1007/s00134-017-4698-z 28204860

[38] Wang XT, Peng Z, An YY, Shang T, Xiao G, He S, Chen X, Zhang H, Wang Y, Wang T, Zhang JH, Gao X, Zhu Y, Feng Y. Paeoniflorin and Hydroxysafflor Yellow A in Xuebijing Injection Attenuate Sepsis-Induced Cardiac Dysfunction and Inhibit Proinflammatory Cytokine Production. Front Pharmacol. 2021; 11:614024. 10.3389/fphar.2020.614024 33986658 PMC8112230

[39] Aengevaeren VL, Baggish AL, Chung EH, George K, Kleiven Ø, Mingels AMA, Ørn S, Shave RE, Thompson PD, Eijsvogels TMH. Exercise-Induced Cardiac Troponin Elevations: From Underlying Mechanisms to Clinical Relevance. Circulation. 2021; 144:1955–72. 10.1161/CIRCULATIONAHA.121.056208 34898243 PMC8663527

[40] Maeder M, Fehr T, Rickli H, Ammann P. Sepsis-associated myocardial dysfunction: diagnostic and prognostic impact of cardiac troponins and natriuretic peptides. Chest. 2006; 129:1349–66. 10.1378/chest.129.5.1349 16685029

[41] Aakre KM, Saenger AK, Body R, Collinson P, Hammarsten O, Jaffe AS, Kavsak P, Omland T, Ordonez-Lianos J, Apple FS. Analytical Considerations in Deriving 99th Percentile Upper Reference Limits for High-Sensitivity Cardiac Troponin Assays: Educational Recommendations from the IFCC Committee on Clinical Application of Cardiac Bio-Markers. Clin Chem. 2022; 68:1022–30. 10.1093/clinchem/hvac092 35716089

[42] Wang X, Qin W, Qiu X, Cao J, Liu D, Sun B. A novel role of exogenous carbon monoxide on protecting cardiac function and improving survival against sepsis via mitochondrial energetic metabolism pathway. Int J Biol Sci. 2014; 10:777–88. 10.7150/ijbs.9220 25076854 PMC4115198

[43] Wang X, Xie D, Dai H, Ye J, Liu Y, Fei A. Clemastine protects against sepsis-induced myocardial injury *in vivo* and *in vitro*. Bioengineered. 2022; 13:7134–46. 10.1080/21655979.2022.2047256 35274595 PMC9208445

[44] Zhao L, Zhao H, Sun M, Chen M, Wu X, Deng C, Yang W, Tian Y, Wang Q, Liang Z, Xu X, Yang Y. Kudzu Celery Decoction Exerts Protection against Sepsis-Induced Myocardial Injury. Oxid Med Cell Longev. 2022; 2022:2886932. 10.1155/2022/2886932 35571240 PMC9095356

[45] Liu C, Zou Q, Tang H, Liu J, Zhang S, Fan C, Zhang J, Liu R, Liu Y, Liu R, Zhao Y, Wu Q, Qi Z, Shen Y. Melanin nanoparticles alleviate sepsis-induced myocardial injury by suppressing ferroptosis and inflammation. Bioact Mater. 2022; 24:313–21. 10.1016/j.bioactmat.2022.12.026 36632502 PMC9813528

[46] Shi C, Wang X, Wang L, Meng Q, Guo D, Chen L, Dai M, Wang G, Cooney R, Luo J. A nanotrap improves survival in severe sepsis by attenuating hyperinflammation. Nat Commun. 2020; 11:3384. 10.1038/s41467-020-17153-0 32636379 PMC7341815

[47] Zhai GH, Zhang W, Xiang Z, He LZ, Wang WW, Wu J, Shang AQ. Diagnostic Value of sIL-2R, TNF-α and PCT for Sepsis Infection in Patients With Closed Abdominal Injury Complicated With Severe Multiple Abdominal Injuries. Front Immunol. 2021; 12:741268. 10.3389/fimmu.2021.741268 34745113 PMC8569904

[48] Deng P, Tang N, Li L, Zou G, Xu Y, Liu Z. Diagnostic value of combined detection of IL-1β, IL-6, and TNF-α for sepsis-induced cardiomyopathy. Med Clin (Barc). 2022; 158:413–7. 10.1016/j.medcli.2021.04.025 34147250

[49] Wu Y, Zhao M, Lin Z. Pyrroloquinoline quinone (PQQ) alleviated sepsis-induced acute liver injury, inflammation, oxidative stress and cell apoptosis by downregulating CUL3 expression. Bioengineered. 2021; 12:2459–68. 10.1080/21655979.2021.1935136 34227919 PMC8806920

[50] Cao YY, Wang Z, Wang ZH, Jiang XG, Lu WH. Inhibition of miR-155 alleviates sepsis-induced inflammation and intestinal barrier dysfunction by inactivating NF-κB signaling. Int Immunopharmacol. 2021; 90:107218. 10.1016/j.intimp.2020.107218 33296782

[51] Lin H, Chen H, Qi B, Jiang Y, Lian N, Zhuang X, Yu Y. Brain-derived extracellular vesicles mediated coagulopathy, inflammation and apoptosis after sepsis. Thromb Res. 2021; 207:85–95. 10.1016/j.thromres.2021.09.014 34583153

[52] An R, Zhao L, Xi C, Li H, Shen G, Liu H, Zhang S, Sun L. Melatonin attenuates sepsis-induced cardiac dysfunction via a PI3K/Akt-dependent mechanism. Basic Res Cardiol. 2016; 111:8. 10.1007/s00395-015-0526-1 26671026

[53] Jenner A, Peña-Blanco A, Salvador-Gallego R, Ugarte-Uribe B, Zollo C, Ganief T, Bierlmeier J, Mund M, Lee JE, Ries J, Schwarzer D, Macek B, Garcia-Saez AJ. DRP1 interacts directly with BAX to induce its activation and apoptosis. EMBO J. 2022; 41:e108587. 10.15252/embj.2021108587 35023587 PMC9016351

[54] Ansari MY, Ball HC, Wase SJ, Novak K, Haqqi TM. Lysosomal dysfunction in osteoarthritis and aged cartilage triggers apoptosis in chondrocytes through BAX mediated release of Cytochrome c. Osteoarthritis Cartilage. 2021; 29:100–12. 10.1016/j.joca.2020.08.014 33161099 PMC8418332

[55] Czabotar PE, Lessene G, Strasser A, Adams JM. Control of apoptosis by the BCL-2 protein family: implications for physiology and therapy. Nat Rev Mol Cell Biol. 2014; 15:49–63. 10.1038/nrm3722 24355989

[56] Porter AG, Jänicke RU. Emerging roles of caspase-3 in apoptosis. Cell Death Differ. 1999; 6:99–104. 10.1038/sj.cdd.4400476 10200555

[57] Araya LE, Soni IV, Hardy JA, Julien O. Deorphanizing Caspase-3 and Caspase-9 Substrates In and Out of Apoptosis with Deep Substrate Profiling. ACS Chem Biol. 2021; 16:2280–96. 10.1021/acschembio.1c00456 34553588 PMC9116730

[58] Kepp O, Rajalingam K, Kimmig S, Rudel T. Bak and Bax are non-redundant during infection- and DNA damage-induced apoptosis. EMBO J. 2007; 26:825–34. 10.1038/sj.emboj.7601533 17235284 PMC1794390

[59] Virág L, Robaszkiewicz A, Rodriguez-Vargas JM, Oliver FJ. Poly(ADP-ribose) signaling in cell death. Mol Aspects Med. 2013; 34:1153–67. 10.1016/j.mam.2013.01.007 23416893

[60] Han X, Chen D, Liufu N, Ji F, Zeng Q, Yao W, Cao M. MG53 Protects against Sepsis-Induced Myocardial Dysfunction by Upregulating Peroxisome Proliferator-Activated Receptor-α. Oxid Med Cell Longev. 2020; 2020:7413693. 10.1155/2020/7413693 32908637 PMC7474382

[61] Efferth T, Oesch F. The immunosuppressive activity of artemisinin-type drugs towards inflammatory and autoimmune diseases. Med Res Rev. 2021; 41:3023–61. 10.1002/med.21842 34288018

[62] Zhou Q, Tian W, Jiang Z, Huang T, Ge C, Liu T, Zhao F, Chen T, Cui Y, Li H, Yao M, Li J, Tian H. A Positive Feedback Loop of AKR1C3-Mediated Activation of NF-κB and STAT3 Facilitates Proliferation and Metastasis in Hepatocellular Carcinoma. Cancer Res. 2021; 81:1361–74. 10.1158/0008-5472.CAN-20-2480 33361392

[63] Li S, Han S, Jin K, Yu T, Chen H, Zhou X, Tan Z, Zhang G. SOCS2 Suppresses Inflammation and Apoptosis during NASH Progression through Limiting NF-κB Activation in Macrophages. Int J Biol Sci. 2021; 17:4165–75. 10.7150/ijbs.63889 34803490 PMC8579457

[64] Yu H, Lin L, Zhang Z, Zhang H, Hu H. Targeting NF-κB pathway for the therapy of diseases: mechanism and clinical study. Signal Transduct Target Ther. 2020; 5:209. 10.1038/s41392-020-00312-6 32958760 PMC7506548

[65] Zhang X, Su C, Zhao S, Li J, Yu F. Combination therapy of Ulinastatin with Thrombomodulin alleviates endotoxin (LPS) - induced liver and kidney injury via inhibiting apoptosis, oxidative stress and HMGB1/TLR4/NF-κB pathway. Bioengineered. 2022; 13:2951–70. 10.1080/21655979.2021.2024686 35148668 PMC8973693

[66] Hwang SJ, Wang JH, Lee JS, Kang JY, Baek DC, Kim GH, Ahn YC, Son CG. Ginseng Sprouts Attenuate Mortality and Systemic Inflammation by Modulating TLR4/NF-κB Signaling in an LPS-Induced Mouse Model of Sepsis. Int J Mol Sci. 2023; 24:1583. 10.3390/ijms24021583 36675101 PMC9860726

[67] Kucharczak J, Simmons MJ, Fan Y, Gélinas C. To be, or not to be: NF-kappaB is the answer--role of Rel/NF-kappaB in the regulation of apoptosis. Oncogene. 2003; 22:8961–82. 10.1038/sj.onc.1207230 14663476

[68] Chen XS, Cui JR, Meng XL, Wang SH, Wei W, Gao YL, Shou ST, Liu YC, Chai YF. Angiotensin-(1-7) ameliorates sepsis-induced cardiomyopathy by alleviating inflammatory response and mitochondrial damage through the NF-κB and MAPK pathways. J Transl Med. 2023; 21:2. 10.1186/s12967-022-03842-5 36593471 PMC9807106

[69] Lu YC, Yeh WC, Ohashi PS. LPS/TLR4 signal transduction pathway. Cytokine. 2008; 42:145–51. 10.1016/j.cyto.2008.01.006 18304834

[70] Chen SN, Tan Y, Xiao XC, Li Q, Wu Q, Peng YY, Ren J, Dong ML. Deletion of TLR4 attenuates lipopolysaccharide-induced acute liver injury by inhibiting inflammation and apoptosis. Acta Pharmacol Sin. 2021; 42:1610–9. 10.1038/s41401-020-00597-x 33495514 PMC8463538

[71] Xiao Z, Kong B, Fang J, Qin T, Dai C, Shuai W, Huang H. Ferrostatin-1 alleviates lipopolysaccharide-induced cardiac dysfunction. Bioengineered. 2021; 12:9367–76. 10.1080/21655979.2021.2001913 34787054 PMC8809987

[72] Jia F, Liu Y, Dou X, Du C, Mao T, Liu X. Liensinine Inhibits Osteosarcoma Growth by ROS-Mediated Suppression of the JAK2/STAT3 Signaling Pathway. Oxid Med Cell Longev. 2022; 2022:8245614. 10.1155/2022/8245614 35116094 PMC8807040

[73] Chen Y, Shao Z, Jiang E, Zhou X, Wang L, Wang H, Luo X, Chen Q, Liu K, Shang Z. CCL21/CCR7 interaction promotes EMT and enhances the stemness of OSCC via a JAK2/STAT3 signaling pathway. J Cell Physiol. 2020; 235:5995–6009. 10.1002/jcp.29525 32017846

[74] Yu L, Zhang Y, Chen Q, He Y, Zhou H, Wan H, Yang J. Formononetin protects against inflammation associated with cerebral ischemia-reperfusion injury in rats by targeting the JAK2/STAT3 signaling pathway. Biomed Pharmacother. 2022; 149:112836. 10.1016/j.biopha.2022.112836 35339827

[75] Kim YK, Lee JY, Suh HN. Cytokine-Induced JAK2-STAT3 Activates Tissue Regeneration under Systemic or Local Inflammation. Int J Mol Sci. 2022; 23:2262. 10.3390/ijms23042262 35216377 PMC8877378

[76] Tan Z, Liu Q, Chen H, Zhang Z, Wang Q, Mu Y, Li Y, Hu T, Yang Y, Yan X. Pectolinarigenin alleviated septic acute kidney injury via inhibiting Jak2/Stat3 signaling and mitochondria dysfunction. Biomed Pharmacother. 2023; 159:114286. 10.1016/j.biopha.2023.114286 36706631

[77] Zanders L, Kny M, Hahn A, Schmidt S, Wundersitz S, Todiras M, Lahmann I, Bandyopadhyay A, Wollersheim T, Kaderali L, Luft FC, Birchmeier C, Weber-Carstens S, Fielitz J. Sepsis induces interleukin 6, gp130/JAK2/STAT3, and muscle wasting. J Cachexia Sarcopenia Muscle. 2022; 13:713–27. 10.1002/jcsm.12867 34821076 PMC8818599

[78] Stark GR, Darnell JE Jr. The JAK-STAT pathway at twenty. Immunity. 2012; 36:503–14. 10.1016/j.immuni.2012.03.013 22520844 PMC3909993

[79] Xu S, Pan X, Mao L, Pan H, Xu W, Hu Y, Yu X, Chen Z, Qian S, Ye Y, Huang Y, Pan J. Phospho-Tyr705 of STAT3 is a therapeutic target for sepsis through regulating inflammation and coagulation. Cell Commun Signal. 2020; 18:104. 10.1186/s12964-020-00603-z 32641132 PMC7341624

